# Effect of Processing Techniques on the Microstructure and Mechanical Performance of High-Density Polyethylene

**DOI:** 10.3390/polym13193346

**Published:** 2021-09-29

**Authors:** Edgar Mejia, Nizamudeen Cherupurakal, Abdel-Hamid I. Mourad, Sultan Al Hassanieh, Mohamed Rabia

**Affiliations:** 1 Mechanical Engineering Department, College of Engineering, United Arab Emirate University, Al Ain 15551, United Arab Emirates; emejia8@illinois.edu (E.M.); 201890121@uaeu.ac.ae (N.C.); 100049708@ku.ac.ae (S.A.H.); 2 National Eater and Energy Center, United Arab Emirate University, Al Ain 15551, United Arab Emirates; 3 Mechanical Design Department, Faculty of Engineering, Helwan University, Cairo 11795, Egypt; 4 Polymer Research Laboratory, Chemistry Department, Faculty of Science, Beni-Suef University, Beni-Suef 62514, Egypt; mohamedchem@science.bsu.edu.eg

**Keywords:** high-density polyethylene, injection molding, compression molding, microstructure, mechanical properties

## Abstract

The versatility of high-density polyethylene (HDPE) makes it one of the most used polymers for vast applications ranging from food packaging to human implants. However, there still is confusion regarding the proper selection of processing techniques to produce HDPE specimens for high-end applications. Herein, we compare the processing of HDPE by two relevant techniques: compression and injection molding. The fabricated samples were studied using uniaxial tensile testing to determine their mechanical performance. Furthermore, the microstructure of samples was analyzed using different characterization techniques. Compression-molded specimens recorded a higher degree of crystallinity (DC) using two different characterization techniques such as differential scanning calorimetry (DSC) and X-ray diffraction (XRD). With this information, critical processing factors were determined, and a general structure–property relationship was established. It was demonstrated that having a higher DC resulted in higher yield strength and Young’s modulus. Furthermore, premature failure was observed in the injection-molded specimens, resulting in lower mechanical performance. This premature failure was caused due to flow marks observed using scanning electron microscopy (SEM). Therefore, it is concluded that compression molding produces superior samples compared to injection molding.

## 1. Introduction

Plastics have become indispensable in today’s world as their uses are innumerable, ranging from everyday items, such as food containers and furniture, to highly specialized items, such as heart valves and military helmets. The myriad of applications of polymers drives researchers to constantly develop various manufacturing processes that accommodate different thermal, mechanical, and chemical property requirements. Polyethylene with general formula (C_2_H_4_)n is one of the most commonly used polymers due to its chemical inertness, low cost, and strength [1,2,3,4,5]. Properties of polyethylene vary significantly due to the number of different chain architectures and molecular weights and are highly dependent on the synthesis process. The most ubiquitous variants of polyethylene and its derivatives are polytetrafluoroethylene (PTFE), low-density polyethylene (LDPE), high-density polyethylene (HDPE), and ultra-high molecular weight polyethylene (UHWWPE) [6,7,8,9]. Among these, HDPE has received worldwide attention due to its unique properties such as high strength to weight ratio, high impact resistance, durability, moldability, and weather resistance. Thus, the application of HDPE is found everywhere, starting from plastic containers, toys, to automobile parts to enhance fuel efficiency [10,11].

Polyethylene is a thermoplastic, making traditional thermoforming processes such as injection molding and compression molding common in processing. Both techniques increase the polymer’s temperature above its melting point (130–137 °C), allowing the polymer to flow and take the mold’s shape while cooling [12,13,14,15]. The melted polymer is forced through a metallic die for injection molding, filling the mold with a rapid pressure increase [16,17,18]. On the other hand, the polymer is held under static pressure for compression molding. These two processes result in samples with different mechanical behaviors and properties because of the varying molecular interactions among the chains, which result in intricate hierarchal microstructures [19,20,21,22]. Although research works on investigating the relationships between the mechanical properties of polymers and their microstructure are numerous [23,24,25,26], few works exist that directly compare the effects of the various thermoforming process on the resulting polymer samples.

The effects of varying temperatures on the microstructure, morphology, and chain mobility of HDPE were investigated by Hedesiu et al. It was reported that the method in which HDPE is processed highly impacts the material’s final mechanical and thermal characteristics [27]. The experiments performed in this study established the temperature ranges in which the most significant differences in molecular mobility were observed for the semi-rigid crystal–amorphous interface. The soft fraction of the amorphous phase—this analysis provided accurate determination of the phase composition and the thicknesses of the domains mentioned above. Furthermore, they showed the three-phase model that best describes the phase composition of HDPE. The results are in good agreement with mechanisms previously proposed by Bureau et al. regarding partial melting and surface crystallinity upon the annealing of HDPE and pre-melting temperatures [28].

Bureau et al. investigated and compared the phase morphology of polymer blends between HDPE and polystyrene (PS) using injection molding and compression molding. This work also concisely discusses the mechanical characterization of pure HDPE, stating that injection molding causes anisotropic behavior, contrary to the homogenous behavior exhibited by the compression-molded HDPE [29]. They found that the transverse and longitudinal injection-molded samples have ultimate stress higher than compression molding. Furthermore, samples prepared by both manufacturing methods show similar failure strain values regardless of orientation. However, contradicting results were presented by Xie while utilizing ultra-high molecular weight polyethylene (UHMWPE) [30]. This work stated that the failure strains of the samples fabricated by compression molding are twice those obtained by injection molding. The results prompted an investigation into the morphology of the samples, and it was concluded that a brittle failure in the samples causes the isotropic skin–core structure. Thus, it becomes necessary to conduct a deeper analysis of the morphology resulting from the two fabrication methods.

This paper will thoroughly investigate the effects of both injection molding and compression molding on the thermo-mechanical properties of pure high-density polyethylene (HDPE). The structure–property relationships of both fabrication methods will also be analyzed. The results of this study are highly significant to manufacturers making decisions regarding which fabrication method is most suitable to their needs.

## 2. Materials and Methods

The high-density polyethylene (HDPE) used in this study was obtained from Sigma-Aldrich, Missouri, MO, USA (serial number: 547999) with a melt index of 2.2 g/10 min.

### 2.1. Sample Preparation

The raw material used for both injection molding and compression molding was pure HDPE pellets. A 100 mm by 100 mm mold was fabricated for compression molding to prepare HDPE sheets with 2 mm thickness. The pellets were loaded into the mold and placed in a double-plate vertical hot press. The parameters of temperature, time, and pressure were maintained at 150 °C, 15 min, and 18 kN, respectively. Once the process was completed, the compression-molded sample was left to cool down to room temperature. The pellets were fed into the injection molding barrel and quickly injected into a rectangular mold with a thickness of 2 mm. The parameters such as barrel temperatures (*T_h_*), injected at pressure (*I_p_*), barrel hold time (*t_b_*), mold hold time (*t_m_*), and mold temperature (*T_m_*) were maintained constant at *T_h_* = 160 °C. *I_p_* = 8 bar, *t_b_* = 15 min, *t_m_* = 30 s, and *T_m_* =100 °C, respectively. The optimized parameters used were obtained from the previous works [31]. The rectangular samples produced had dimensions of 2.5 cm by 7.5 cm. Then, both the injection and compression-molded samples were cut into a dumbbell shape using a punching die. The die has a 20 mm gauge length, a width of 4 mm, and an average thickness of 2 mm, following ISO37-type 3 standard. Further details on the fabrication process may be found in A-H.I. Mourad et.al. [5,32,33].

### 2.2. Mechanical Testing

The tensile tests were performed on an MTS universal testing machine equipped with a load cell of 5 KN. A crosshead speed of 25 mm/min was chosen for reasons explained in the following sections. Mechanical properties such as yield strength, fracture stress, modulus, and strain at break were obtained from the stress–strain curves [10,34,35,36,37,38]. These results were obtained by averaging at least five specimens with a standard deviation of less than 5%. The fracture stresses and failure strains were the largest values obtained from all five test samples, following the assumptions that all the other samples had failed prematurely.

The creep behavior of the injection and compression-molded samples were evaluated on the creep measurement apparatus SM106. The specimen was held at one end to a static holder using a pin, and the other side was connected to a leverage mechanism. Loads were applied by hanging weights of 1 kg at the lever’s opposite end of the sample. The deformation is captured using a camera and evaluated over time for plotting.

### 2.3. Morphological Analysis

A thermogravimetric analysis (TGA) test was carried out using a Q-50 Thermogravimetric analyzer (TA Instruments Inc., Newark, DE, USA) to evaluate the thermal stability of HDPE samples. The sample was heated in a controlled atmosphere with a heating rate of 10 °C/min until reaching 600 °C, under a flow of N_2_ gas at a rate of 60 mL/min. The weight history was plotted to assess the thermal stability of HDPE at each temperature [39,40].

Similarly, Differential Scanning Calorimetry (DSC) tests were performed on TA-Instruments (DSC Q200) with a 5–10 mg specimen weight to obtain melting temperature and percentage of crystallization. The specimens were heated up to 200 °C at 10 °C/min in a nitrogen environment to obviate specimen oxidation. The enthalpy of fusion obtained from the DSC was used to calculate the degree of crystallinity (DC). A total of 290 J/g was taken as the theoretical heat of fusion of an HDPE sample with a 100% degree of crystallinity [41].

To validate the DC obtained from DSC, X-ray diffraction (XRD) was performed. Equation (1), below, was utilized to calculate the DC, as explained in Aggarwal and Tilley’s work [42].

(1) $$DC=\frac{I_{110}+1.41*I_{200}}{I_{110}+1.41*I_{110}+0.75*I_{am}}*100\%$$

Finally, the morphological analysis at different synthesis conditions was studied using a scanning electron microscope (SEM) JEOL- JSM 7610F at different magnifications.

## 3. Results and Discussion

### 3.1. Strain-Rate Testing

**Figure 1:**
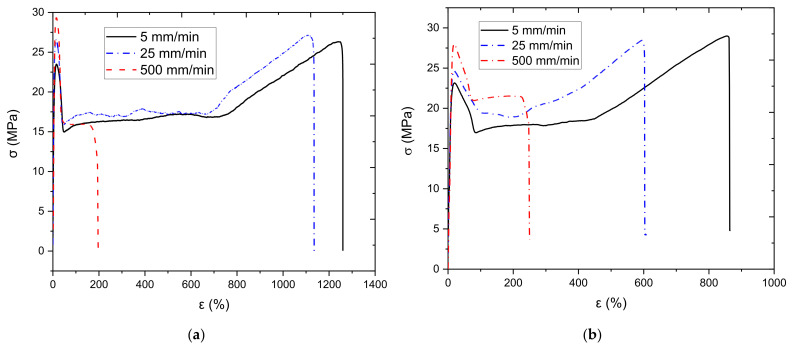
Tensile test at different test speeds: (**a**) compression-molded sample, (**b**) injection-molded sample.

Tensile tests at various test speeds were conducted to investigate the effect of strain rate on the mechanical properties of obtained samples and to identify the optimal testing speed for future tests. The compression-molded and injection-molded samples were tested under three different test speeds of 5 mm/min, 25 mm/min, and 500 mm/min. The results from the testing are illustrated below in Figure 1, and the data can be found in Table 1. Similar trends are observed for both molded samples, where the modulus and yield stress increase with strain rate. However, the failure strain is decreased dramatically at a certain threshold. This behavior is expected and well established, as the polymer chain mobility decreases with increasing strain rates. As the strain rate increases, polymer chains have little time to respond to the external load and untangle and realign, giving rise to a more brittle response, which is the behavior exhibit by both types of samples. More information regarding this phenomenon can be found in [43,44].

Figure 1 a,b show that the sample’s behavior remains virtually identical, and their mechanical properties show only minor variations at test speeds of 5 mm/min and 25 mm/min. However, the time it takes to run the test at 5 mm/min is more than five times longer than the time it would take to run the test at 25 mm/min. Consequently, it was decided to perform all the following tests in the paper at 25 mm/min. It is critical to mention that the failure strain of the injection-molding sample at 500 mm/min exceeds that of the compression-molded sample. In contrast, the compression-molded sample has a higher failure strain at the two other test speeds. This phenomenon will be discussed in the coming sections.

### 3.2. Tensile Testing

**Figure 2:**
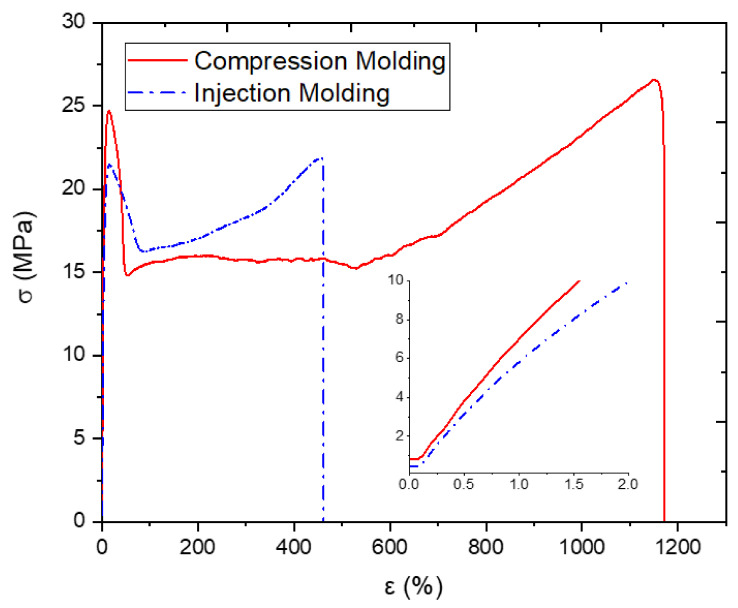
Compression and injection-molded samples’ stress–strain curves.

The mechanical behaviors of samples fabricated by both compression and injection molding will first be investigated. Figure 2 shows the tensile tests conducted on the samples processed using injection molding and compression molding. This figure also shows an insert of the same data at low strain values, less than 2% strain, to better observe the difference in elastic modulus. The mechanical response of both samples may be divided into four distinct deformation phases.

**Figure 3:**
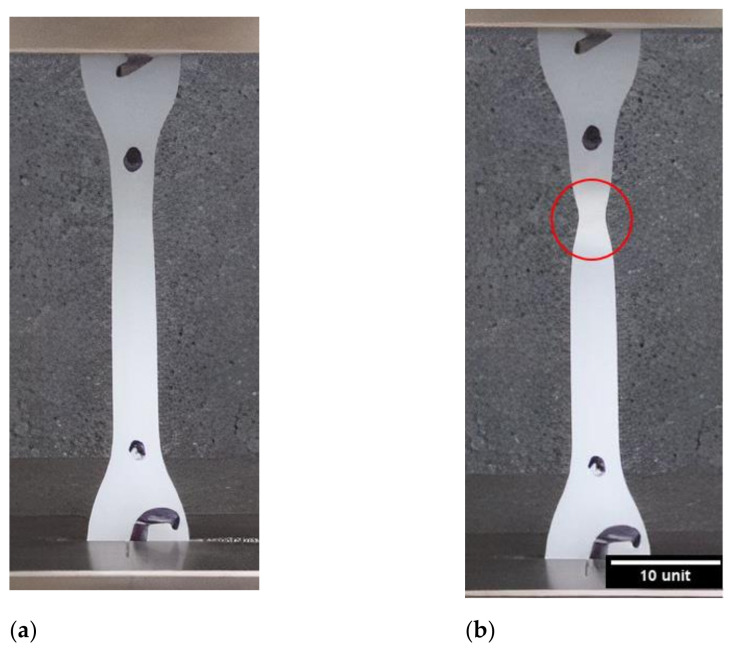
(**a**) Initial state of the tested sample, (**b**) start of the necking phenomenon during tensile testing.

The 1st phase, present in both samples, is a linear elastic response that can be observed in the insert of the graph. The initial increase reaches a maximum stress value, and then, the graph decreases. This first peak on the stress–strain curve will be taken as the yield strength of the material. The 2nd phase follows shows a steep drop in the stress–strain curve, which is attributed to a local reduction in the cross-sectional area of the gauge length—a phenomenon referred to as necking. The necking phenomenon is illustrated in Figure 3b. The 3rd distinct region is characterized by an increase in strain with a minimal fluctuation in stress, creating a plateau. During this stage, the necked region propagates along the gauge length of the sample due to the disentanglement and alignment of the polymer chains. Once the amorphous segments of the polymer are aligned, the crystalline segments will keep elongating and develop into micro-fibrils. This process is also known as plastic deformation, creating what could be considered a secondary specimen. The 4th stage shows a toughening in the response of the HDPE samples due to the effect of cold drawing. A rise in the stress–strain curve is seen until the sample fractures.

**Figure 4:**
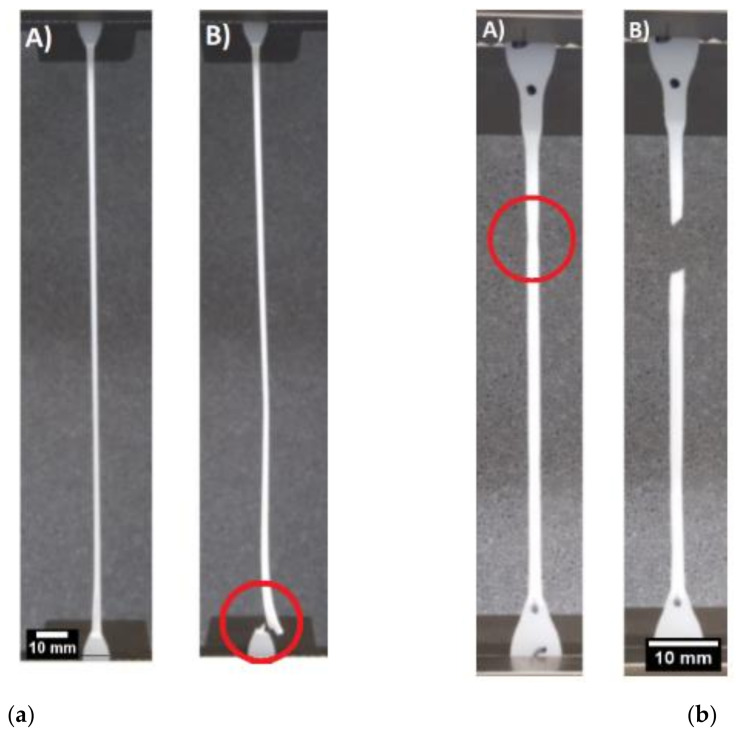
Distinct failure modes: (**a**) compression-molded sample, (**b**) injection-molded sample.

As illustrated in Figure 4, the samples fabricated using injection molding and compression molding exhibit different failure modes. The compression-molded sample continuously elongates until the necked region engulfs the entirety of the gauge length and reaches the shoulder of the sample, to which failure occurs at the junction of the drawn polymer and the undrawn section, as illustrated in Figure 4a. However, in Figure 4b, a second local thinning may arise in the injection-molded sample, causing sudden failure. As shown in Table 2, the mechanical properties of the compression-molded samples are superior to those obtained by the injection-molded samples. The compression-molded samples show a failure strain twice as large as that of the injection-molded sample. In addition, the elastic modulus and yield strength of the compression molded sample are approximately 10% higher than those in the injection-molded sample. This disparity will be investigated and elaborated on in the following section through a detailed morphological analysis. Furthermore, the mechanical properties from various previous studies on HDPE were also tabulated in Table 2. Compression-molded HDPE, as reported by Amjadi and Fatemi [45], has a modulus higher than that of all of the injection-molded samples. Furthermore, Amjadi and Fatemi [45] also reported that the tensile strength of compression-molded HDPE is larger than that of injection-molded HDPE. Other published results are presented to compare with the obtained results in this experiment. Tisserat et al. showed similar results for yield stress, and Mararis et al. show a similar modulus for pristine HDPE [46,47]. It is important to mention that there could be some variation between all the results due to different molecular weight (grade), processing parameters, and testing speed.

### 3.3. Morphological Analysis

**Figure 5:**
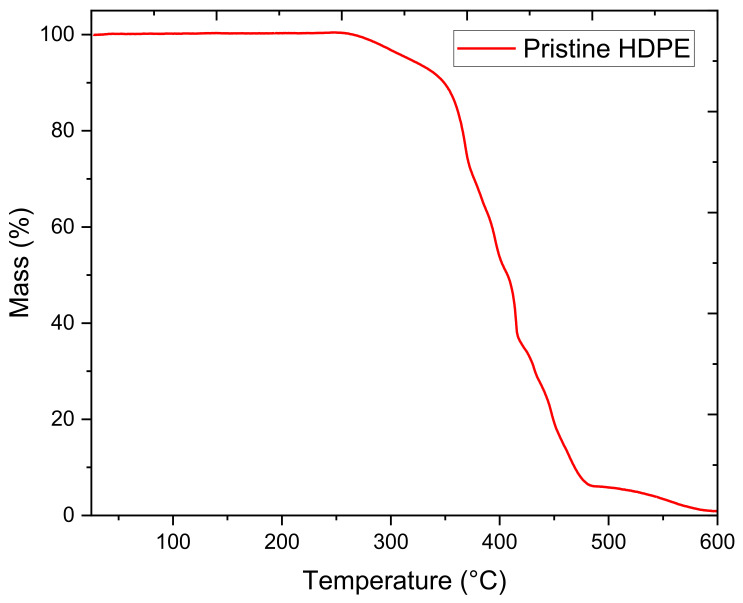
Thermal results of pristine HDPE obtained by TGA.

Before performing any thermal analysis, a TGA analysis was conducted to understand HDPE’s thermal stability. The results of the TGA analysis are shown in Figure 5 below. As illustrated in the figure, HDPE is stable until a temperature of approximately 270 °C, where evident thermal degradation occurs. After a sudden drop in mass, the decrease in mass becomes constant from 360 to 480 °C. Finally, the polymer reaches a mass of zero at around 600 °C. Consequently, an upper limit of 270 °C was established for any thermal analysis, assuring the thermal stability of the samples.

Digital scanning calorimetry (DSC) analysis was performed on the samples fabricated using both processes. To obtain the degree of crystallinity, the measured heat of fusion (∆H) was divided by the heat of fusion of a theoretical HDPE sample with 100% crystallinity, with ∆H of 290 J/kg. The heat of fusion represents the amount of energy required to melt the sample. It is well established that crystalline sections of a polymer require higher amounts of energy to melt. Therefore, the change of enthalpy of 232.4 J/g found in the compression-molded sample represents a higher degree of crystallinity than the one obtained for injection molding. The results obtained from the DSC analysis are tabulated and compared with the previous studies in Table 3 below.

As a direct result of having a higher degree of crystallinity, the compression-molded sample presents a higher modulus of elasticity and higher yield stress. Hedesiu [27] reported that if the samples are allowed to cool down slowly, the molecular chains of polymer can organize themselves into an organized structure, leading to a higher degree of crystallinity. Furthermore, the larger static pressure held using the compression-molding process causes the structure to pack closer, reducing the number of voids in the microstructure. In similar studies conducted by Vijay et al. [48] of compression-molded HDPE, the melting temperature was found to be 132 °C, and the degree of crystallinity was found to be 78.73%. The studies of Lorena et al. [49] and Sotomayor et al. [50] on injection-molded HDPE found the melting peaks at 134.8°C and 130.8 °C, respectively. Furthermore, the degree of crystallinity was also less compared to the compression-molded samples.

XRD testing was conducted to confirm the results and conclusions made from the DSC analysis. The results obtained from the XRD analysis reaffirmed that the compression-molded samples possess a higher degree of crystallinity than the injection-molded samples. For the compression-molded sample, the DC was 88.71% and 83.29% for the injection-molded sample. The nominal value for the difference in the degree of crystallinity obtained by the DSC analysis is very close to that obtained by the XRD analysis, 5.3 and 5.0, respectively. The minor difference may be attributed to the fact that XRD provides information from the sample’s surface, while the results provided by the DSC analysis are from the core of the sample.

**Figure 6:**
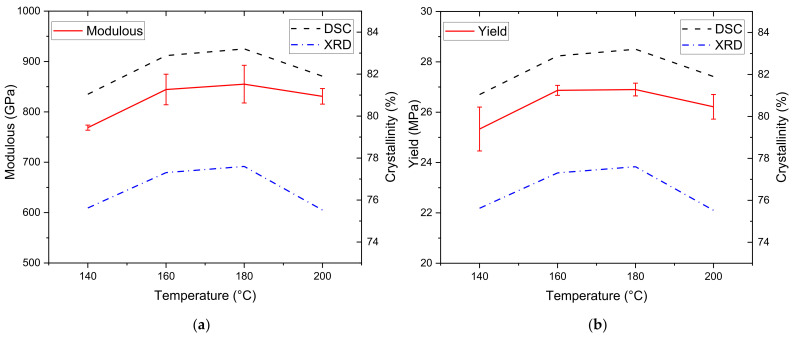
Crystallinity vs. mechanical properties: (**a**) modulus, (**b**) yield strength.

The correlation between the mechanical properties of HDPE and the degree of crystallinity is further illustrated in Figure 6. The Young’s modulus and degree of crystallization are plotted against the temperature change, and a similar graph is constructed for the yield stresses. This analysis was done by varying the processing temperature of the samples and then using DSC and XRD to determine the degree of crystallinity for only compression-molded samples. The correlation becomes evident and shows that the degree of crystallinity obtained from both DSC and XRD increases along with the specimen’s modulus and yield stress.

### 3.4. Structure–Property Relationship

**Figure 7:**
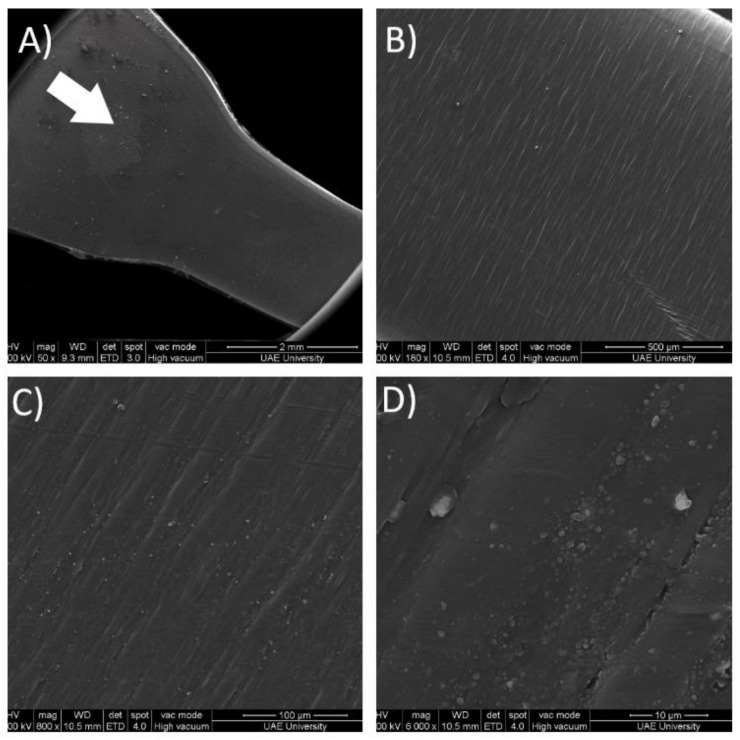
SEM imagining for injection-molded sample at different magnifications. (**A**) 50×, (**B**) 180×, (**C**) 800× and (**D**) 6000×.

It is remarkable that though the compression-molded sample has a higher degree of crystallinity, it exhibits a more ductile behavior. To address this, SEM imaging was performed, and the results are illustrated in Figure 7. Figure 7A shows the junction between the shoulder of the dumbbell-shaped injection-molded sample and the gauge length. The white arrow on Figure 7A indicates the direction of the filling procedure, and the orientation is maintained in all the subsequent images. As the magnification increases, surface defects known as flow marks start becoming more and more evident.

These surface defects are well characterized by Gan-Ji Zhong [32]. Flow marks appear due to uneven cooling at the interface between the polymer and the mold as the polymer flows into the cavity, which explains why the defects are perpendicular to the flow direction. These defects are also perpendicular to the loading direction and act as stress raisers when the load is applied, causing the injection-molded sample to be weaker than its compression-molded counterpart. The defects may also explain the second necking region that causes premature failure. Thus, the relationship between the manufacturing processes, the morphology, and the mechanical properties obtained has been established, and an investigation into the mechanical properties in a broader fashion will ensue.

### 3.5. Creep Testing

The effects of strain rate on the behavior of both samples have been discussed above. It is noteworthy to mention that although the compression-molded sample’s failure strain is higher at test speeds of 5 mm/min and 25 mm/min, that is not the case at 500 mm/min. This phenomenon may be explicable by the molecular structure of the two samples. It has been previously established that compression-molded samples have a higher degree of crystallinity than injection-molded samples, so it may be the case that at high testing speeds, the polymer chains found in the injection-molded samples untangle faster, giving it a more ductile response.

**Figure 8:**
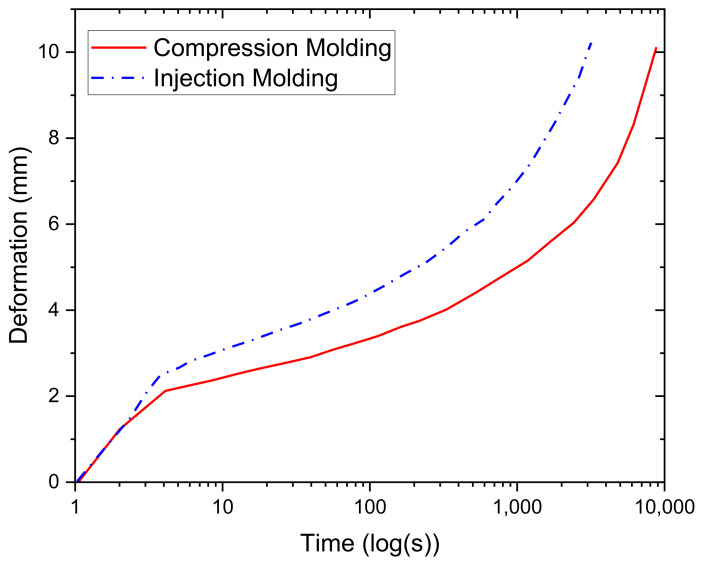
Creep testing for both compression-molded and injection-molded samples.

To better understand this, creep testing was also performed on both the compression-molded and injection-molded samples to provide a comparative analysis of both samples’ behaviors. The creep test results are illustrated in Figure 8; however, it is essential to mention that the samples did not fail due to the testing setup’s limitations. The creep test results show that the instantaneous deformation of the injection-molded sample is more significant than that of the compression-molded sample, which is to be expected due to its lower stiffness for the reasons mentioned in the above discussions. The compression-molded samples have a longer creep time, which may be attributed to the larger degree of crystallinity. The crystalline regions tend to delay the creep behavior. It is hypothesized that this phenomenon occurs due to the lower mobility of the polymer chain in the crystalline regions, which causes the compression-molded sample to see less deformation than the injection-molded sample at any given point in time. This explanation has also been suggested by Sakai [51].

## 4. Conclusions

This study performed a comparative analysis and characterization of the mechanical properties of injection-molded and compression-molded samples. A clear relationship between the microstructure of the HDPE samples and their mechanical performance was found and thoroughly investigated. However, more work needs to be done to characterize further and understand the relationship between the processing parameters, microstructure, and properties. Two major conclusions were made in this study and are summarized below.

- The first is that compression-molded samples have superior mechanical properties due to the higher degree of crystallinity and compact packing.
- The premature failure observed in the injection-molded samples are caused by defects that arise due to the flow of the polymer during filling.

## Figures and Tables

**Table 1:** Compression-molded and injection-molded sample mechanical properties.

Processing	Velocity	Modulus	Yield Stress	Tensile Strength	Failure Strain
	(mm/min)	(MPa)	(MPa)	(MPa)	(%)
Compression-molded	5	524	23.49	26.32	1259.97
	25	647	26.57	27.12	1134.96
	500	455	29.35	15.93	197.01
Injection-molded	5	339.15	23.15	28.99	859.72
	25	447.36	24.67	28.44	595.19
	500	322.45	28.01	21.49	249.47

**Table 2:** Compression-molded and injection-molded sample mechanical properties.

Processing	Modulus	Yield Stress	Tensile Strength	Failure Strain
	(MPa)	(MPa)	(MPa)	(%)
Compression-molded Current study	754	24.5	26.6	1170
Injection-molded Current study	672	22.3	21.9	462
Compression-molded Amjadi and Fatemi [45]	810	-	18	-
Injection-molded Amjadi and Fatemi [45]	790	-	17	-
Injection-molded Tisserat et al. [46]	339	21.5	-	105
Injection-molded Morais et al. [47]	543	-	18	-

**Table 3:** DSC results for both processing methods.

Processing	Melting Peak	Change in Enthalpy	Degree of Crystallinity
	(°C)	(J/g)	(%)
Compression-molded Current study	123.32	232.4	80.15
Injection-molded Current study	128.86	217.0	74.84
Compression molding Vijay et al. [48]	132.32	216.72	78.73
Injection molding Lorena et al. [49]	134.8	221.6	75.5
Injection molding Sotomayor et al. [50]	130.8	178.6	61

## Data Availability

All data and models used during the study appear in the submitted article.

## References

[1] Mourad A.-H.I., Dehbi A. (2014). On use of trilayer low density polyethylene greenhouse cover as substitute for monolayer cover. Plast. Rubber Compos..

[2] Mourad A.-H.I., Akkad R.O., Soliman A.A., Madkour T.M. (2009). Characterisation of thermally treated and untreated polyethylene–polypropylene blends using DSC, TGA and IR techniques. Plast. Rubber Compos..

[3] Dehbi A., Mourad A.-H.I., Bouaza A. (2012). Degradation assessment of LDPE multilayer films used as a greenhouse cover: Natural and artificial aging impacts. J. Appl. Polym. Sci..

[4] Mourad A.-H.I., Elsayed H.F., Barton D.C., Kenawy M., Abdel-Latif L.A. (2003). Ultra high molecular weight polyethylene deformation and fracture behaviour as a function of high strain rate and triaxial state of stress. Int. J. Fract..

[5] Babaghayou M.I., Mourad A.-H.I., Lorenzo V., de la Orden M.U., Urreaga J.M., Chabira S.F., Sebaa M. (2016). Photodegradation characterization and heterogeneity evaluation of the exposed and unexposed faces of stabilized and unstabilized LDPE films. Mater. Des..

[6] Nath S., Bodhak S., Basu B. (2008). HDPE-Al2O3-HAp composites for biomedical applications: Processing and characterizations. J. Biomed. Mater. Res. Part B Appl. Biomater..

[7] Mourad A.-H.I., Mozumder M.S., Mairpady A., Pervez H., Kannuri U.M. (2017). On the injection molding processing parameters of HDPE-TiO2 nanocomposites. Materials.

[8] Benabid F.Z., Kharchi N., Zouai F., Mourad A.-H.I., Benachour D. (2019). Impact of co-mixing technique and surface modification of ZnO nanoparticles using stearic acid on their dispersion into HDPE to produce HDPE/ZnO nanocomposites. Polym. Compos..

[9] Mozumder M.S., Mourad A.-H.I., Mairpady A., Pervez H., Haque M.E. (2018). Effect of TiO2 nanofiller concentration on the mechanical, thermal and biological properties of HDPE/TiO2 nanocomposites. J. Mater. Eng. Perform..

[10] Mejia E.B., Al-Maqdi S., Alkaabi M., Alhammadi A., Alkaabi M., Cherupurakal N., Mourad A.-H.I. Upcycling of HDPE waste using additive manufacturing: Feasibility and challenges. In Proceedings of the IEEE 2020 Advances in Science and Engineering Technology International Conferences (ASET).

[11] Pervez H., Mozumder M.S., Mourad A.-H.I. (2016). Optimization of injection molding parameters for HDPE/TiO_2_ nanocomposites fabrication with multiple performance characteristics using the Taguchi method and grey relational analysis. Materials.

[12] Huang L., Wang Z., Zheng G., Guo J.Z., Dai K., Liu C. (2015). Enhancing oriented crystals in injection-molded HDPE through introduction of pre-shear. Mater. Des..

[13] Bhatti M.M., Abdelsalam S.I. (2021). Thermodynamic entropy of a magnetized Ree-Eyring particle-fluid motion with irreversibility process: A mathematical paradigm. ZAMM.

[14] Raza R., Mabood F., Naz R., Abdelsalam S.I. (2021). Thermal transport of radiative Williamson fluid over stretchable curved surface. Therm. Sci. Eng. Prog..

[15] Eldesoky I.M., Abdelsalam S.I., El-Askary W.A., Ahmed M.M. (2020). The integrated thermal effect in conjunction with slip conditions on peristaltically induced particle-fluid transport in a catheterized pipe. J. Porous Media.

[16] Abdelsalam S.I., Velasco-Hernández J.X., Zaher A.Z. (2021). Electro-magnetically modulated self-propulsion of swimming sperms via cervical canal. Biomech. Model. Mechanobiol..

[17] Elkoumy S.R., Barakat E.I., Abdelsalam S.I. (2013). Hall and transverse magnetic field effects on peristaltic flow of a Maxwell fluid through a porous medium. Glob. J. Pure Appl. Math..

[18] Abdelsalam S.I., Zaher A.Z. (2021). Leveraging elasticity to uncover the role of rabinowitsch suspension through a wavelike conduit: Consolidated blood suspension application. Mathematics.

[19] Dikobe D.G., Luyt A.S. (2017). Thermal and mechanical properties of PP/HDPE/wood powder and MAPP/HDPE/wood powder polymer blend composites. Thermochim. Acta.

[20] Mourad A.-H.I., Idrisi A.H., Wrage M.C., Abdel-Magid B.M. (2019). Long-term durability of thermoset composites in seawater environment. Compos. Part B Eng..

[21] Dehbi A., Mourad A.-H.I. (2016). Durability of mono-layer versus tri-layers LDPE films used as greenhouse cover: Comparative study. Arab. J. Chem..

[22] Dehbi A., Mourad A.-H.I., Bouaza A. (2011). Ageing effect on the properties of tri-layer polyethylene film used as greenhouse roof. Procedia Eng..

[23] Ferhoum R., Aberkane M., Hachour K. (2014). Analysis of thermal ageing effect (hold time-crystallinity rate-mechanical property) on high density polyethylene (HDPE). Int. J. Mater. Sci. Appl..

[24] LEE D.-J. (2006). Comparison of mechanical properties of compression and injection molded PEEK/Carbon fiber reinforced composites. Key Eng. Mater..

[25] Ghiam F., White J.L. (1991). Phase morphology of injection-molded blends of nylon-6 and polyethylene and comparison with compression molding. Polym. Eng. Sci..

[26] Bledzki A.K., Faruk O. (2004). Wood fiber reinforced polypropylene composites: Compression and injection molding process. Polym. Technol. Eng..

[27] Hedesiu C., Demco D.E., Kleppinger R., Buda A.A., Blümich B., Remerie K., Litvinov V.M. (2007). The effect of temperature and annealing on the phase composition, molecular mobility and the thickness of domains in high-density polyethylene. Polymer.

[28] Kodjie S.L., Li L., Li B., Cai W., Li C.Y., Keating M. (2006). Morphology and crystallization behavior of HDPE/CNT nanocomposite. J. Macromol. Sci. Part B Phys..

[29] Bureau M.N., El Kadi H., Denault J., Dickson J.I. (1997). Injection and compression molding of polystyrene/high-density polyethylene blends? Phase morphology and tensile behavior. Polym. Eng. Sci..

[30] Xie M., Chen J., Li H. (2009). Morphology and mechanical properties of injection-molded ultrahigh molecular weight polyethylene/polypropylene blends and comparison with compression molding. J. Appl. Polym. Sci..

[31] Mozumder M.S., Mourad A.-H.I., Perinpanayagam H., Zhu J. (2014). NanoTiO_2_-Enriched Biocompatible Polymeric Powder Coatings: Adhesion, Thermal and Biological Characterizations. Trans. Tech. Publ..

[32] Zhong G.-J., Li Z.-M. (2005). Injection molding-induced morphology of thermoplastic polymer blends. Polym. Eng. Sci..

[33] Mourad A.-H.I., Fouad H., Elleithy R. (2009). Impact of some environmental conditions on the tensile, creep-recovery, relaxation, melting and crystallinity behaviour of UHMWPE-GUR 410-medical grade. Mater. Des..

[34] Mourad A.-H.I. (2010). Thermo-mechanical characteristics of thermally aged polyethylene/polypropylene blends. Mater. Des..

[35] Idrisi A.H., Mourad A.-H.I., Abdel-Magid B.M., Shivamurty B. (2021). Investigation on the durability of E-Glass/Epoxy composite exposed to seawater at elevated temperature. Polymers.

[36] Idrisi A.H., Mourad A.-H.I., Sherif M.M. (2021). Impact of prolonged exposure of eleven years to hot seawater on the degradation of a thermoset composite. Polymers.

[37] Mourad A.-H.I., Maiti S.K. (1995). Influence of state of stress on mixed mode stable crack growth through D16AT aluminium alloy. Int. J. Fract..

[38] Mourad A.-H.I., Idrisi A.H., Christy J.V., Thekkuden D.T., Al Jassmi H., Ghazal A.M., Syam M.M., Ali Ahmed Al Qadi O.D. (2019). Mechanical performance assessment of internally-defected materials manufactured using additive manufacturing technology. J. Manuf. Mater. Process..

[39] Mourad A.-H.I., Cherupurakal N., Hafeez F., Barsoum I., Genena F.A., Al Mansoori M.S., Al Marzooqi L.A. (2020). Impact strengthening of laminated kevlar/epoxy composites by nanoparticle reinforcement. Polymers.

[40] Babaghayou M.I., Mourad A.-H.I., Ochoa A., Beltrán F., Cherupurakal N. (2020). Study on the thermal stability of stabilized and unstabilized low-density polyethylene films. Polym. Bull..

[41] Wunderlich B. (1973). Macromolecular Physics.

[42] Aggarwal S.L., Tilley G.P. (1955). Determination of crystallinity in polyethylene by X-ray diffractometer. J. Polym. Sci..

[43] Jang B.Z., Uhlmann D.R., Sande J.B. (1984). Vander Ductile–brittle transition in polymers. J. Appl. Polym. Sci..

[44] Li Z., Lambros J. (2001). Strain rate effects on the thermomechanical behavior of polymers. Int. J. Solids Struct..

[45] Amjadi M., Fatemi A. (2020). Tensile behavior of high-density polyethylene including the effects of processing technique, thickness, temperature, and strain rate. Polymers.

[46] Tisserat B., Reifschneider L., Joshee N., Finkenstadt V.L. (2013). Properties of high density polyethylene–Paulownia wood flour composites via injection molding. BioResources.

[47] De Morais J.A., Gadioli R., De Paoli M.-A. (2016). Curaua fiber reinforced high-density polyethylene composites: Effect of impact modifier and fiber loading. Polímeros.

[48] Vijay A.R.M., Ratnam C.T., Khalid M., Appadu S., Gupta T. (2020). Effect of radiation on the mechanical, morphological and thermal properties of HDPE/rPTFE blends. Radiat. Phys. Chem..

[49] Amoroso L., Heeley E.L., Ramadas S.N., McNally T. (2020). Crystallisation behaviour of composites of HDPE and MWCNTs: The effect of nanotube dispersion, orientation and polymer deformation. Polymer.

[50] Sotomayor M.E., Krupa I., Várez A., Levenfeld B. (2014). Thermal and mechanical characterization of injection moulded high density polyethylene/paraffin wax blends as phase change materials. Renew. Energy.

[51] Sakai T., Hirai Y., Somiya S. (2018). Estimating the creep behavior of glass-fiber-reinforced polyamide considering the effects of crystallinity and fiber volume fraction. Mech. Adv. Mater. Mod. Process..

